# Effectiveness of group-based indicated prevention in children identified with disruptive behavior problems: results of an implementation study in the German health care system

**DOI:** 10.1186/s13034-025-00902-8

**Published:** 2025-05-16

**Authors:** Maria McDonald, Theresia Fippel, Susanne Knappe, Patricia Theresa Porst, Cornelia Beate Siegmund, Julia Zink, Max Weniger, Katja Beesdo-Baum, Veit Roessner

**Affiliations:** 1 Department of Child and Adolescent Psychiatry and Psychotherapy, Faculty of Medicine, TUD Dresden University of Technology, Fetscherstr 74, 01307 Dresden, Germany; 2 Behavioral Epidemiology, Institute of Clinical Psychology and Psychotherapy, TUD Dresden University of Technology, Dresden, Germany; 3 Evangelische Hochschule Dresden (Ehs), University of Applied Sciences for Social Work, Education and Nursing, Dresden, Germany

**Keywords:** Disruptive behavior problems, Childhood, Indicated prevention, Mental health

## Abstract

**Background:**

Subclinical disruptive behavior problems often occur during childhood and are a risk factor for developing a mental disorder later in life. To prevent a manifestation of dysfunctional disruptive behavior, early intervention is critical. This study aimed to examine the effectiveness of an indicated prevention program in children with disruptive behavior problems.

**Methods:**

Screening for disruptive behavior problems was conducted using the Strength and Difficulties Questionnaire during routine pediatric health check-ups. Depending on their risk status (normal vs. borderline vs. abnormal), children received a recommendation for no intervention, an indicated prevention program (i.a. “Baghira”) or further diagnostics. Questionnaires such as the Child Behavior Checklist and the Parent Rating Scale for Conduct Disorder (DISYPS Competence scale) were administered at three time points (T0: pre-intervention, T1: 6 months after screening/ post-intervention, T2: 6 months after T1). Children who participated in “Baghira” (BA *n* = 171), a cognitive-behavioral group program for children with disruptive behaviors, were compared to children screened as normal (NOR *n* = 881) or received a recommendation for “Baghira” but refused participation (NO BA *n* = 46).

**Results:**

Disruptive behavior problems decreased (BA: β = − 3.61, p <.001) and prosocial behavior increased (BA: β = 1.67, p <.001) in the BA compared to the NOR group from T0 to T1. These effects were maintained at T2 follow-up (BA: β = − 1.60; p =.035; β = 1.12; p =.019). However, the NO BA group also improved in prosocial behavior and from T0 to T1.

**Conclusion:**

Although an improvement in disruptive behavior symptoms as well as an increase in prosocial behavior were observed, controlled studies using matched or stratified designs are needed to replicate the effectiveness of “Baghira” in a prevention context, apart from the Covid-19 pandemic, to improve children’s mental health.

**Supplementary Information:**

The online version contains supplementary material available at 10.1186/s13034-025-00902-8.

## Introduction

Mental health problems in children and adolescents are one of the greatest challenges facing society today. For example, data from the German *KIGGS study* (2014–2017) revealed current mental health problems in 16.9% of 3–17-year-olds [1]. Many of these mental health problems are subclinical, such as emotional and disruptive behavior problems, and pose a risk for the development of mental disorders. Longitudinal studies have shown that a significant proportion of such cases becomes clinically relevant [2–4]. Both subclinical emotional and disruptive behavior problems and mental disorders, pose a serious threat to the children’s quality of life and further development [5], generate high costs for the health care system [6, 7] and are therefore of great societal relevance.

In the following, we use the term “disruptive behavior problems” to refer to this type of behavior, which combines aggressive, externalizing and oppositional behavior. Such behavior often emerges as early as kindergarten and can remain stable over time [8, 9]. However, not every socially undesirable behavior like occasional tantrums, aggression, stubbornness, or clashes with peers or family should be classified as a behavioral problem, because these are often age-dependent parts of normative child development [10]. However, disruptive behavior should receive greater attention (e.g. early detection of symptoms and, if necessary, treatment) if it occurs with atypical frequency or severity for the child's age, affects multiple life domains, and is associated with social impairment [11]. For instance, children with behavioral problems, particularly those displaying disruptive behavior problems that violate social norms and involve oppositional conduct, often experience short-term negative consequences, such as rejection and exclusion by peers. Additionally, they experience long-term negative consequences, such as reduced (health-related) quality of life and an increased risk of developing a mental disorder [12–16]. When left untreated, significant impairment can be the consequence negatively affecting developmental trajectories [2, 17]. For instance, establishing cooperative social relationships is one of the critical developmental milestones for preschoolers [18]. When disruptive behavior problems become chronic, they may evolve into disorders such as oppositional defiant disorder (ODD) and conduct disorder (CD) [19]. The long-term consequences of these disorders include risky alcohol consumption, illicit drug use, delinquency, and a propensity to violence [20]. Early interventions, in the form of indicated prevention programs, could presumably prevent these developmental trajectories from leading to the clinical manifestation of a mental disorder [21]. Moreover, research shows that such early interventions are more cost-effective in the long run than later interventions [22]. Such programs could focus on helping children to improve their ability to categorize emotions like fear, anger, or sadness in younger children [23] which are linked to the child’s social behavior and are associated with disruptive behavior.

Generally, different strategies for prevention include universal (targeting the general population), selective (targeting individuals with increased biological, social, or psychological risk for a future disorder) and indicated (symptoms of a disorder that are not (yet) sufficient to meet the required criteria for a disorder) prevention [24]. In German-speaking countries, several universal prevention programs for children focus on topics such as social skill training, empathy development, and conflict management [25, 26]. So far, they have shown smaller effect sizes for disruptive behavior problems compared to indicated prevention programs [27, 28]. Furthermore, compared to universal prevention, indicated prevention programs were more cost-effective in children and adults [29]. Also, child-centered prevention programs for disruptive behavior problems have not been well evaluated in controlled trials for reducing, e.g. aggressive behaviors (but see [30]). In a clinical context in German speaking countries, disorder-specific, child-centered programs such as the *Treatment Program for Children with Aggressive Behavior* (THAV; [31–33] or the *Baghira Group Training* [34] for children with oppositional defiant disorder/conduct disorder have been evaluated, e.g. in a randomized controlled trial with moderate treatment effects [32]. These programs may also be applicable for indicated prevention [21].

One approach to determine whether a child might benefit from support through an indicated prevention program for disruptive behavior problems is to use a brief screening instrument, such as the Strengths and Difficulties Questionnaire (SDQ; [35]). The latter can be incorporated in a care chain from screening to prevention services. To maximize outreach, the screening could be integrated into routine pediatric health check-ups therein creating an innovative and efficacious care chain [36, 37], as in Germany pediatric health services are regularly used by the majority of children aged 3–10 years. Beyond identifying basic preventive needs, it is essential to allocate each child to a program that is specifically tailored to unfold any preventive effect or, ideally, the most effective one available. The informative and non-tailored nature of universal prevention may not allow the full potential of preventive effects to unfold in young children, as compared to the tailored support provided through indicated prevention. One such program that offers this support for children with already present disruptive behavior problems could be the *Baghira Group Training* which addresses topics related to emotion recognition and regulation—both considered to contribute to the persistence of disruptive behavior problems [23, 38]. This program is recommended for children aged 8–13 years [34]. The *Baghira Group Training* could serve as an indicated prevention program for disruptive behavior problems, as it aims to provide alternatives to disruptive behavior while promoting socially competent behaviors [34]. This training has previously been evaluated in combination with the *Positive Parenting Program Triple P* [39] in a clinical setting [40]. In the group that received a combined treatment with the *Baghira Group Training* and *Triple P*, disruptive behavior problems in the parent ratings along with rule-breaking behaviors in the teachers’ reports significantly decreased compared to those in the waiting list group. In addition, empathy skills improved in the combined treatment group. These effects were sustained at the 6-months follow-up [40].

In general, the effectiveness of an indicated prevention program could be affected by several factors. For example, younger age of the child has been shown to be positively associated with training success [41, 42], as less rigid behaviors in younger children are more malleable compared to older children [43]. Regarding sex, boys tended to display more overtly aggressive behavior than girls [44], often leading to rejection and potentially diminishing their willingness to change their behavior. However, previous studies have shown that the child’s sex does not play a significant role in the effectiveness of an intervention [40, 42].

Probable parental disorder, however, was associated with poorer treatment responses in the child’s behavior: Parents with mental disorders were less able to provide sufficient emotional availability and support to continuously attend sessions [45–47]. Parental stress is another important factor in shaping an environment that supports a child’s therapeutic progress, as high levels of parental stress are linked to poorer treatment outcome for the child [48, 49]. Furthermore, parent motivation has been associated with parental disorder and has been shown to play an important role in treatment participation, with higher levels of parent motivation leading to better treatment outcomes [50, 51]. Notably, however, this relationship has not been explored in the context of prevention.

We hypothesized that participation in the indicated prevention program “Baghira” (BA) would result in a reduction in disruptive behavior and an increase in prosocial behavior. Therefore, we focused on prosocial behavior and disruptive behavior specific measures. We expected the BA group to become more similar to children without disruptive behavior problems (NOR). In contrast, we hypothesized that families who refused participation (NO BA) would, on average, show no change in disruptive behavior problems over time in their parents view. Additionally, we assumed that the NOR group would also not change in their behavior. To clarify the potential preventive effect of participating in “Baghira”, we further hypothesized that a probable parental disorder, lower levels of parental stress, higher levels of parental motivation, as well as higher/lower child age would predict improvements of disruptive behavior problems. Finally, we did not expect that child sex would predict symptom improvement in the BA group.

## Methods

### Procedure

The PROMPt project, a screening and prevention implementation study (October 2018–September 2022), was conducted in Dresden, Germany [37]. 46 pediatricians participated in the project to screen children during regular routine pediatric health check-ups (U9–U11, typically aged 5–10 years). Details on the participating pediatricians can be found in Weniger et al. [52]. The categorization of the original SDQ score [53] was slightly adapted to ensure a broader range to identify children with emotional and disruptive behavior problems and further reach families and their children who could profit from a participation in an indicated prevention program (see Supplementary S1 for categorization). Group assignments for examining the program’s effectiveness were based on these project-specific adaptations and are consistent with previous publications on this topic [54, 55].

The child’s legal guardians were asked to fill out a project-specific questionnaire that included questions regarding socio-demographic information such as family status or monthly household income (after taxes) but also barriers to the utilization of health care services. They also filled out the SDQ and later received feedback from their pediatrician regarding possible emotional and disruptive behavior problems and, where appropriate, a recommendation for participation in a prevention program. This included cases where SDQ scores were elevated and pediatricians could recommend program participation if they considered it beneficial. Children with emotional problems were recommended the program “Mutig werden mit Til Tiger” (Becoming brave with Til Tiger”, short: Til Tiger; [56], for analyses regarding emotional problems see [55]), those with disruptive behavior problems received a recommendation for participation in “Baghira”, based on the *Baghira Group Training* [34]. Families interested in participation were then asked to contact the PROMPt study team directly. If they did not do so, but had given written informed consent at the pediatrician's office to be contacted by the project team, they were approached up to five times by the study team via phone or email after approximately 3–4 weeks to inquire about their interest. There were two access routes: through screening at the pediatricians’ office or families contacting the study team on their own (e.g. based on a recommendation from friends). Regardless of the referral pathway, the same criteria for study inclusion applied, families received the same study information, and were subsequently invited to on-site initial interview with the family, administering the SDQ screening and during which a psychologist (project team member) reviewed the indication for possible participation. Participation in the prevention program was only possible if the child gave verbal assent and all legal guardians gave written consent to participate. Exclusion criteria for participation were a reported diagnosed clinical disorder according to ICD-10 in the disruptive behavior or emotional spectrum within the past 6 months, unstable medication, current psychotherapy, or acute self-harm or danger to others. In case of *abnormal SDQ-*scores, parents were advised to consult a psychologist/psychotherapist/psychiatrist for further diagnostic procedures or counseling services. Families participating in the indicated prevention programs (i.e. “Baghira” or “Til Tiger”) who completed the questionnaires received 10€ per assessment (30€ in total for completing all three measurement points). Families from other groups (e.g. NOR or NO BA group) who completed the questionnaires were entered into a drawing where board games were given as prizes. With this paper, we focus on the indicated prevention program “Baghira”, for children with disruptive behavior problems.

### Participants

**Fig. 1:**
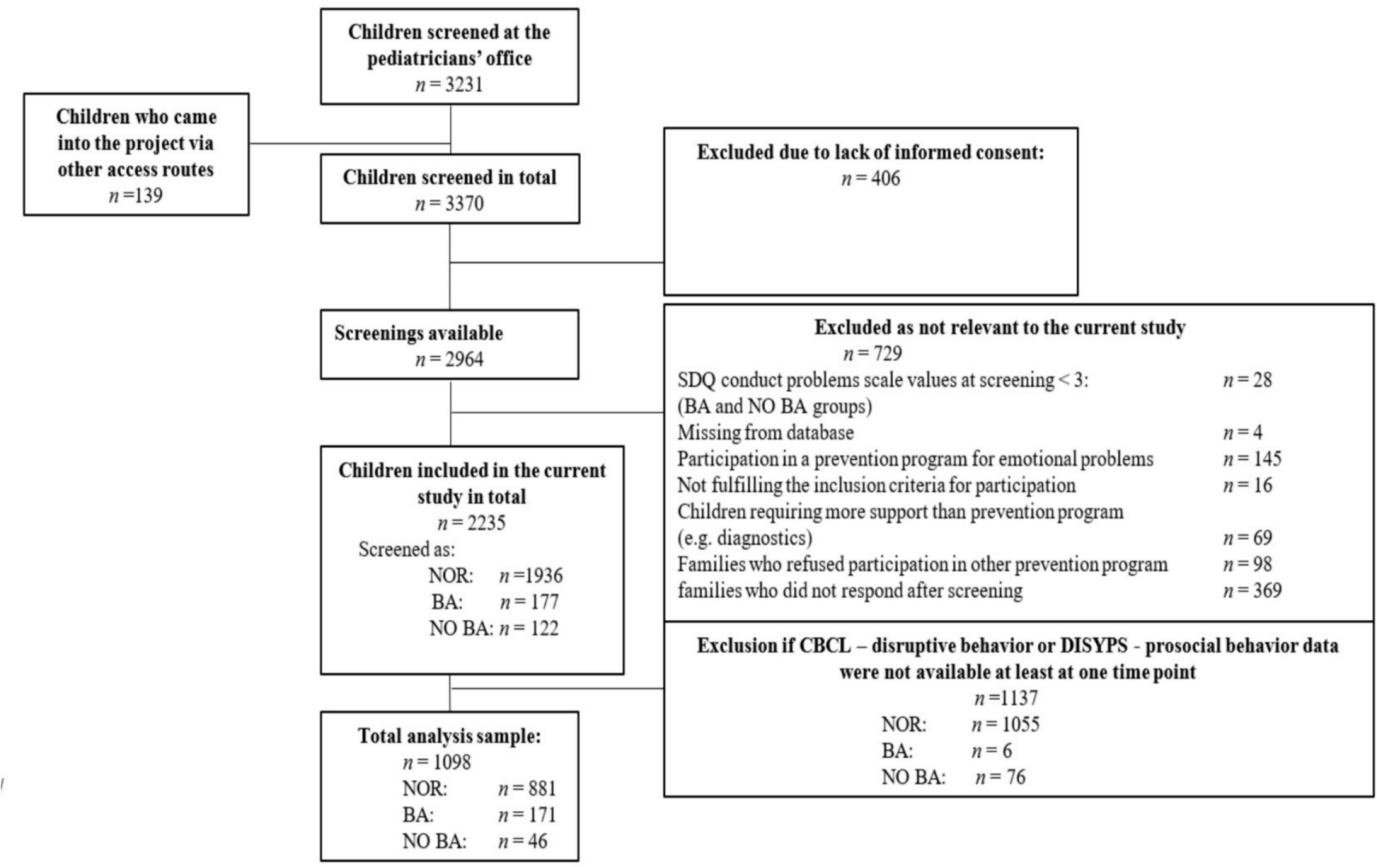
Flowchart of the analysis sample. *N* = number of participants; Screenings according to the SDQ [53] in the pediatrician’s offices: *NOR* children screened as normal, *BA* children who received the recommendation for the prevention program “Baghira" and participated for at least one session in the program, *NO BA* children who were recommended to participate in “Baghira” but refused to participate

In total, *n* = 3231 children were screened during regular health check-ups at pediatric practices in the Dresden area (Germany) or the families registered themselves for a screening (*n* = 139) with a member of the PROMPt-project team. As the present screening and prevention study was implemented in a naturalistic health care setting, sample size estimates were based on expected service availability and utilization rates (for details, see [37]). Of those screened children, *n* = 406 were excluded from the analyses due to a lack of written informed consent for screening or participation in a prevention program from their legal guardians. Further exclusions were made for cases that were not relevant to the current study (*n* = 729), such as a recommendation for participation in the other indicated prevention program or refusal to participate. Six children recommended for and participated in both programs; of these, three were included as they first participated in “Baghira”. Further exclusions occurred when parents did not complete the relevant questionnaires at least at one time point (*n* = 1137; see Fig. 1 for details).

The following analyses are based on data that refer to participants who completed the questionnaires CBCL [57, 58] and DISYPS-prosocial behavior (competence scale [59]) for at least one time point and had an SDQ score ≥ 3 in conduct problems scale if they received a recommendation for participation in “Baghira” either from their pediatrician or after the on-site initial interview with a member of the study team. Children with abnormal SDQ scores were not excluded if the pediatrician determined them as eligible for indicated prevention (*n* = 33). *N* = 881 children were screened as ‘normal’ (NOR), *n* = 171 children received a recommendation for a prevention program and took part in “Baghira” (BA) and *n* = 46 children refused participation despite receiving a recommendation for “Baghira” from their pediatrician or a member of the study team (NO BA; see S3 for given reasons). On average, *M* = 8.27 (*SD* = 1.03; Range: 2–9 sessions) sessions were attended by children in the BA group (91.93%). Drop-out after one or more “Baghira” sessions was generally low (*n* = 5 children).

### Prevention program “Baghira”

The training used in the current study is based on the *Baghira Group training* developed by Aebi and colleagues [34]. This cognitive behavioral group program was originally designed for clinical use with children exhibiting oppositional and aggressive behavior. It has been evaluated in both inpatient and outpatient settings [34] and has shown long-term positive effects [40]. In the PROMPt project, “Baghira” consisted of nine weekly sessions of 90 min each conducted in groups of three to five children. The program’s playful nature—including games and the main character, a panther named Baghira—makes it adaptable even for children under 8 years old. For the PROMPt project, materials were adapted to fit a prevention context and younger children. For instance, the original session duration of 120 min was reduced to 90 min, incorporating a short break. We predominantly used visual materials of the Baghira training [34], especially to account for limited reading and writing abilities of participants. As the main components and structure of the training remained unchanged, we do not assume that these modifications affected the current findings. Baghira the panther is introduced during the first session and serves as a guide for the child through the program alongside the trainer. Each session follows the same structure, beginning with a fantasy journey, which serves as a focused relaxation and creates a mindful starting point for the upcoming session. Group rules were reinforced using a token system, rewarding children for adhering to them. Topics covered in the training included emotions and self-awareness, anger and aggression management, impulse and anger control, conflict and problem solving, empathy and perspective taking, as well as positive and negative feedback. The prevention program was conducted by certified and supervised psychology students with at least a bachelor's degree and psychologists who were part of the project team. Regular supervision by a board-certified child and adolescent psychotherapist and project team member ensured fidelity to the program. Parents were informed about the session content after each meeting by the trainer. Additionally, an informational evening was held for the parents, providing information regarding the problem behavior and an opportunity to exchange experiences.

### Measurements

To measure disruptive behavior problems, the sum scores of the subscales aggressive and delinquent behaviors which together form the externalizing behavior scale of the Child Behavior Checklist 4–18 (CBCL; [58]) and the prosocial behavior (competence scale) of the *Parent Rating Scale for Oppositional Defiant and Conduct Disorder* (FBB-SSV), of the Diagnostic System for Mental Disorders in Childhood and Adolescence (DISYPS-III; [59]), were used at time points T0, T1 and T2 (for results regarding the SDQ as a broader measurement for both prevention programs see Siegmund et al. [54, 60]).

### CBCL—disruptive behavior

The CBCL uses a 3-point scale ranging from 0 = not true, 1 = somewhat or sometimes true to 2 = very true or often true. Higher values indicate higher symptom severity. The questionnaire and subscales were found to have robust psychometric properties with a test–retest-reliability of r = 0.83 and an internal consistency of α = 0.93 [61]. The convergent validity has been classified as good to very good [58]. One item included in the questionnaire that refers to schoolchildren was coded as “0” for children who do not yet go to school. Additionally, item 3 contained a wording error resulting in it being treated as missing. In total, 6.9% missing items (2 for the externalizing behavior scale) were, according to the manual of the questionnaire, allowed to calculate sum scores.

### DISYPS—prosocial behavior

Five subscales of the DISYPS use a 4-point scale from 0 = not applicable, 1 = somewhat applicable, 2 = largely applicable to 3 = particularly applicable, part F uses a 5-point scale with the additional option 4 = there were no problems. Prosocial behavior (competence scale) of the DISYPS (4-point scale) was chosen as an outcome for training effectiveness and was administered in all three groups while the complete DISYPS FBB-SSV was only collected from children of the BA group and is only of relevance for the second research question. Analyses for the second research question were restricted to children older than 6 years at T0. Higher values in the competence scale indicate more competence. Sum scores were calculated, with a maximum of 10% missing items allowed. Missing items were coded as 0 “not true” in line with the questionnaire instructions provided by the authors of the German version [59].

### Probable parental disorder, parental stress and motivation

To measure a probable parental disorder, the 9-item Version of the *Symptom Checklist* (SCL-K-9, [62]) and an updated version of the Composite International Diagnostic-Screener (CID-S, [63], were used. The SCL-K-9 was used as a measure of parental psychological distress. The 5-point-scale ranges from 0 = I disagree, 1 = I somewhat disagree, 2 = I partly agree, 3 = I somewhat agree to 4 = I strongly agree. Cronbach's alpha was found to be α = 0.87 and the convergent validity was in the range of r = 0.36 to 0.65 [64].

Another measure of a probable parental disorder, the Composite International Diagnostic-Screener (CID-S; [64]) consists of 12 items for core symptoms of major mental disorders over the past 12 months. Answers regarding the presence of symptoms are provided on a dichotomous scale, and, if applicable, followed by an assessment of the level of burden and treatment utilization. The test–retest reliability was found to be satisfactory (kappa values 0.64–0.92) [65, 66]. A dichotomous variable was computed to indicate a probable disorder, i.e. whether at least one core symptom of a disorder—with associated impairment or burden was present (1) or not (0).

We used the *Parental Motivation Inventory* (PMI, [66]) to measure the parental motivation for a therapeutic intervention. The PMI uses a 4-point scale from 1 = I rather disagree, 2 = I partly agree, 3 = I rather agree to 4 = I fully agree. For the purpose of this study, the PMI was translated into German and some words were adapted to the prevention context. The internal consistency of the PMI is α = 0.96 and the retest reliability is r = 0.76 [67]. For the current sample, the internal consistency is α = 0.91. The PMI, SCL-K-9 and CID-S were only collected at T0 for the BA group.

### Time points and missing data

The PROMPt project used a naturalistic observation study design allowing for differences in subgroup sample sizes and dropout rates. Dropout from screening to subsequent time points occurred and may be due to parents perceiving the screening as part of their pediatrician’s health care and not necessarily with the intention to participate in a study. Data from children participating in “Baghira” were collected on-site within the framework of program participation via tablets whereas data of other participants were collected online using Lime Survey. This could be an explanation for the varying availabilities of questionnaires at the three time points between the groups (see S2 for details). Missing items were treated as random in the analyses. Participants who completed all time points (i.e., completer) and who did not fill in a relevant questionnaire at least at one timepoint (i.e. non-completer) did not differ regarding socio-demographic variables such as monthly income or family status (see S4).

### Statistical analyses

Statistical analyses were performed using SPSS for Windows Version 29.0.0.0 [68] and R [69]. For the current study, we compared children who participated in “Baghira” (BA), who refused participation in “Baghira” (NO BA) as well as children without the necessity of an indicated prevention program (NOR). Data from children of the BA group were included if they attended at least one training session corresponding to ‘intent to treat’. The time period between the time points differed between the three groups but did not vary systematically between the groups in relation to values of the CBCL or DISYPS-prosocial behavior (see S5 and S6). Two generalized linear mixed models (Model 1 and Model 2) with robust estimators and Bonferroni adjusted *p*-values were conducted separately for CBCL and DISYPS-prosocial behavior. Fixed effects included group, time points and interaction between these two factors. In addition, three time points per participant were defined as subject specification. The screening was not included as both measurements, CBCL and DISYPS-prosocial behavior, were not collected during that initial time point. Analyses were conducted separately for the CBCL externalizing behavior scale as a measure for disruptive behavior problems as well as the DISYPS-prosocial behavior of the parent form of the rating scale for Oppositional Defiant and Conduct Disorder (FBB-SSV) which measures prosocial behavior. For both questionnaires, sum scores were used and two separate generalized linear mixed models were performed including the factors group (NOR, BA, NO BA) and time point (T0, T1, T2). The NOR group at T0 was used as reference in Model 1 whereas in Model 2 the NO BA group at T0 served as reference. This approach was applied across both questionnaires. We decided to adopt the linear model for the outcome measures as testing the model with other distributional assumptions did not yield a better model fit in terms of the Akaike Information criterion [70]. Due to violations of the model assumptions, the robust estimation method was used. A priori α-level was set to α = 0.05. Bonferroni correction was applied whenever necessary. Additional exploratory analyses were conducted to further examine the effectiveness of the program. Specifically, we investigated if the effectiveness was moderated by symptom severity as measured by disruptive behavior (CBCL) or prosocial behavior (DISYPS) at T0. For that, children in the BA and NO BA groups were categorized into high (> median) or low (< = median) symptom severity groups based on their respective scores in the questionnaires.

To examine the role of child`s age and sex, as well as probable parental disorder, stress and motivation on training outcome, only the BA group was assed applying two multiple linear regression models. One was conducted for the CBCL—disruptive behavior, the other for the DISYPS—prosocial behavior.

## Results

### Sample characteristics

Results regarding socio-demographic and clinical variables can be found in Table 1. The three groups differed in age, with NO BA group being the youngest (*M*_NO BA_ = 5.96, *SD*_NO BA_ = 1.84), different from both the BA (*p* = 0.03, *M*_Diff_ = − 0.83, 95%-CI [− 1.60 to − 0.06]) and the NOR group (*p* = 0.02, *M*_Diff_ = − 0.79, 95%-CI [− 1.49 to − 0.08]). The proportion of males was higher in both the BA and NO BA group, while the NOR group has a more balanced sex ratio (χ^2^(2) = 57.29 *p* < 0.001). The groups also differed in terms of household income (χ^2^(14) = 28.18 *p* = 0.01). However, the groups did not differ in other socio-demographic data, such as kindergarten or family status.

**Table 1:** Participant characteristics at screening

	Normal (NOR) *n* = 881			Baghira (BA) *n* = 171			No Baghira (NO BA) *n* = 46			Group comparisons		
	*n*	*M*	*SD*	*n*	*M*	*SD*	*n*	*M*	*SD*	Χ^2^/F-test	*df*	*p*
*Age of child (at screening)*	880	6.74	1.95	169	6.79	1.87	46	5.96	1.84	3.74	2	**.024**
	*n*	%		*n*	%		*n*	%				
*Sex of child*	878			171			46					
Female	476	54.2		41	24.0		15	32.6		57.29	2	** <.001**
Male	402	45.8		130	76.0		31	67.4				
*Household monthly* income (after taxes)												
< 1000 €	7	1.1		4	3.2		1	3.8				
1000–2000 €	73	11.1		20	16.1		3	11.5				
2000–3000 €	101	15.4		24	19.4		9	34.6		17.07	8	**.029**
3000–4000 €	249	37.9		42	33.9		5	19.2				
> 4000 €	227	34.6		34	27.4		8	30.8				
Missing	224			47			20					
*Child attending…*												
Kindergarten	367	50.8		63	47.4		21	72.4				
School—First grade	30	4.1		11	8.3		1	3.4				
Second grade	124	17.2		22	16.5		0	–				
Third grade	92	12.7		21	15.8		4	13.8		15.69	10	.109
Fourth grade	70	9.7		8	6.0		1	3.4				
Fifth grade	40	5.5		8	6.0		2	6.9				
*Family status*												
Single parent	70	9.7		20	15.3		6	20.0				
In a relationship/married	627	86.5		108	82.4		23	76.7		6.90	4	.141
Other	28	3.9		3	2.3		1	3.3				

N can vary as not every family filed in all items. Normal: Children that were screened as ‘normal’ according to the SDQ [53], Baghira: Children who received a recommendation from their pediatrician for a prevention program and participated in “Baghira”, No Baghira: Children of families who also received the prevention recommendation from their pediatrician but refused participating in “Baghira”

n: number of cases; M: mean; SD: standard deviation; χ2: Chi square test; df: degrees of freedom. Whenever necessary, Bonferroni correction was used

### Training effectiveness on disruptive behavior problems (CBCL)

**Fig. 2:**
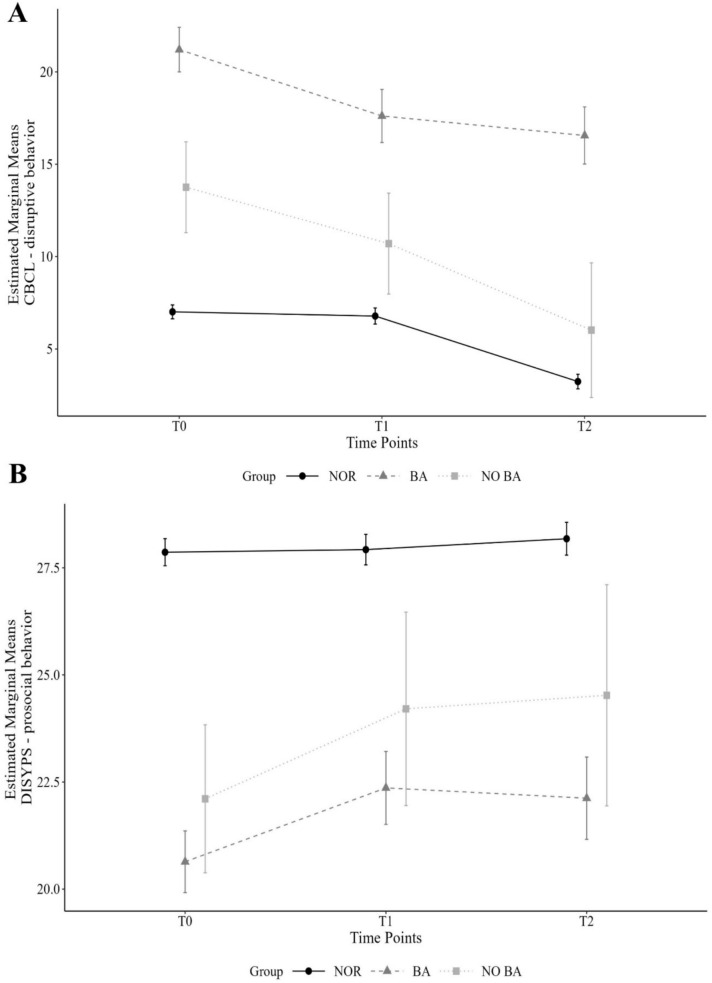
Depicted are estimated marginal means of (**A**) the externalizing behavior scale of the CBCL (disruptive behavior) as well as (**B**) the competence scale of the DISYPS (prosocial behavior) for children screened as normal (NOR), screened to profit from an indicated prevention program “Baghira” and participated in this program (BA) or were screened to profit from the indicated prevention program “Baghira” but refused participation (NO BA). Time points refer to pre-intervention (T0), post-intervention (T1) and 6 months after T1 (T2). Error bars indicate 95% confidence intervals

Results are presented in Fig. 2 and Table 2. The groups differed at T0 (*M*_NOR_ = 7.01, *SD*_NOR_ = 5.18; *M*_BA_ = 21.20, *SD*_BA_ = 7.83; *M*_NO BA_ = 13.76, *SD*_NO BA_ = 7.37; NOR and BA: *p* < 0.001, *M*_Diff_ = − 14.19, 95%-CI [− 15.40 to − 12.99]; BA and NO BA: *p* < 0.001, *M*_Diff_ = 7.45, 95%-CI [4.90–9.99]; NOR and NO BA: *p* < 0.001, *M*_Diff_ = − 6.74, 95%-CI [− 9.10 to − 4.39]) with the BA group initially exhibiting the highest values of disruptive behavior problems in the CBCL. Model 1, which uses NOR as reference, displays main effects for group and time point as well as an interaction effect between the BA group and time points T1 and T2, while also including age and sex of the child as well as monthly household income. Specifically, the BA group showed a decrease in disruptive behavior problems measured by the CBCL from T0 (M = 20.87, SE = 0.68; CI [19.52–22.22]) to T1 (M = 16.96, SE = 0.80; CI [15.36–18.56]; *M*_DiffT0-T1_ = − 3.91 *p* < 0.001, d = 0.60). The decrease from T1 to T2 (M = 15.72, SE = 0.80; CI [14.13–17.32]* M*_DiffT0-T2_ = − 1.24 *p* = 0.274, d = 0.79) was not significant. In contrast, we observed no interaction effect between the NOR x NO BA group at T1 or T2, indicating no difference in terms of change in CBCL—disruptive behavior in the NO BA group when NOR is the reference (NOR: M_T0_ = 7.06, SE_T0_ = 0.21; CI [6.65–7.46]; M_T1_ = 6.76, SE_T1_ = 0.24; CI [6.29–7.23]; M_T2_ = 3.52, SE_T2_ = 0.21; CI [3.11–3.92]; NO BA: M_T0_ = 13.99, SE_T0_ = 1.73; CI [10.59–17.39]; M_T1_ = 12.65, SE_T1_ = 1.76; CI [9.18–16.11]; M_T2_ = 6.99, SE_T2_ = 2.85; CI [1.23–12.75]). For Model 2 that uses NO BA as reference, no interaction effects were detected indicating no differences in symptom change between the groups. Effect sizes (Cohen’s d) for significant interaction effects can be found in Table 2. Additional exploratory analyses revealed no significant three-way interaction between group (BA, NO BA), time (T0, T1, T2) and median group (low vs. high) for disruptive behavior problems. Children with initially higher symptom severity profited more than children with low symptom severity at T1 (see S7 and S8).

**Table 2:** Generalized linear mixed models for the CBCL (disruptive behavior) and the competence scale of the DISYPS (prosocial behavior)

	Disruptive behavior problems CBCL						DISYPS-prosocial behavior					
	*β*	SE	t	*p*	*95% CI*	*d*	*β*	SE	t	*p*	*95% CI*	*d*
*Model 1: NOR as reference*												
Constant	10.30	1.03	9.97	<.001	8.27–12.33		27.48	0.90	30.54	<.001	25.71–29.24	
NOR	–	–	–	–	–		–	–	–	–	–	
BA	13.81	0.72	19.31	<.001	12.40–15.22		− 6.09	0.47	− 13.04	<.001	− 7.01 to − 5.18	
NO BA	6.93	1.74	3.98	<.001	3.50–10.37		− 5.71	0.86	− 6.62	<.001	− 7.41 to − 4.02	
T0	–	–	–	–	–		–	–	–	–	–	
T1	− 0.30	0.17	− 1.78	.076	− 0.63–0.03		0.17	0.14	1.16	.248	− 0.12–0.45	
T2	− 3.54	0.23	− 15.50	<.001	− 3.99 to − 3.09		0.30	0.17	1.77	.077	− 0.03–0.63	
*Interactions*												
BA*T1	− 3.61	0.66	− 5.50	** <.001**	− 4.90 to − 2.32	–0.55	1.67	0.45	3.74	** <.001**	0.79–2.55	0.32
BA*T2	− 1.60	0.76	− 2.12	**.035**	− 3.09 to–0.11	− 0.24	1.12	0.48	2.35	**.019**	0.18–2.06	0.21
NO BA*T1	− 1.05	1.93	− 0.54	.588	− 4.84–2.75		2.08	1.20	1.74	.085	− 0.29–4.45	
NO BA*T2	− 3.46	3.27	− 1.06	.297	− 10.09–3.18		0.73	2.25	0.32	.750	− 3.90–5.35	
Age of child	− 0.56	0.09	− 5.97	<.001	− 0.74 to − 0.38		0.36	0.07	4.80	<.001	0.21–0.51	
Sex of child	1.04	0.35	2.95	.003	0.35–1.73		− 1.71	0.03	− 5.65	<.001	− 2.30 to − 1.12	
Monthly income	− 0.18	0.11	− 1.63	.103	− 0.39–0.04		0.07	0.08	0.83	.406	− 0.09–0.23	
Constant	17.23	1.93	8.94	<.001	13.44–21.02		21.76	1.20	18.29	<.001	19.42–24.10	
NO BA	–	–	–	–	–		–	–	–	–	–	
NOR	− 6.93	1.74	− 3.98	<.001	− 10.37 to − 3.50		5.71	0.89	6.62	<.001	4.02–7.41	
BA	6.90	1.85	3.72	<.001	3.23–10.52		− 0.38	0.94	− 0.40	.689	− 2.24–1.48	
T0	–	–	–	–	–		–	–	–	–	–	
T1	− 1.34	1.92	− 0.70	.485	− 5.12–2.44		2.25	1.19	1.89	.061	− 0.10–4.60	
T2	− 7.00	3.26	− 2.15	.039	− 13.62 to – 0.38		1.02	2.25	0.46	.652	− 3.60–5.63	
*Interactions*												
NOR*T1	1.05	1.93	0.54	.588	− 2.75–4.84		− 2.08	1.20	− 1.74	.085	− 4.45–0.29	
NOR*T2	3.46	3.27	1.06	.297	− 3.18–10.09		− 0.73	2.25	− 0.32	.750	− 5.35–3.90	
BA*T1	− 2.56	2.02	− 1.27	.206	− 6.54–1.42		− 0.41	1.26	− 0.32	.747	− 2.90–2.09	
BA*T2	1.86	3.34	0.56	.582	− 4.91–8.62		0.40	2.29	0.17	.864	− 4.29–5.09	
Age of child	− 0.56	0.09	− 5.97	<.001	− 0.74 to − 0.38		0.36	0.07	4.80	<.001	0.21–0.51	
Sex of child	1.04	0.35	2.95	.003	0.35–1.73		− 1.71	0.03	− 5.65	<.001	− 2.30 to − 1.12	
Monthly income	− 0.18	0.11	− 1.63	.103	− 0.39–0.04		0.07	0.08	0.83	.406	− 0.09–0.23	

*NOR* Children that were screened as ‘normal’ according to the SDQ [53], *BA* Children who participated in “Baghira”, *NO BA* Children of families who refused participating in “Baghira” despite receiving a recommendation. T0: pre-intervention; T1: post-intervention. Regarding child’s sex, females were coded as 1, males as 2. Highlighted in bold are relevant interactions. Robust p-values were used and adjusted for multiple comparisons using Bonferroni correction. Cohen’s d was calculated using the standard deviation of the outcome variable at T0 across all groups (CBCL: SD = 6.54; DISYPS: SD = 5.29). Cohen’s d of 0.2, 0.5 and 0.8 represent small, medium and large effects, respectively [82]

### Training effectiveness on prosocial behavior (DISYPS—prosocial behavior; competence scale)

Results regarding the DISYPS-prosocial behavior are presented in Fig. 2 and Table 2. As for the CBCL, both the BA and NO BA groups differed at time point T0 from the NOR group (*M*_NOR_ = 27.86, *SD*_NOR_ = 4.33; *M*_BA_ = 20.64, *SD*_BA_ = 4.70; *M*_NO BA_ = 22.11, *SD*_NO BA_ = 5.18; NOR and BA: *p* < 0.001, *M*_Diff_ = 7.22, 95%-CI [6.31–8.14]); BA and NO BA: *p* = 0.206, *M*_Diff_ = − 1.47, 95%-CI [− 3.40–0.46]; NOR and NO BA: *p* < 0.001, *M*_Diff_ = 5.75, 95%-CI [3.96–7.55]). For prosocial behavior, the BA group initially had the lowest values. Model 1 (Table 2) displays an interaction effect between NOR x BA group and time points T1 and T2. Estimated marginal means demonstrated an increase in prosocial behavior in the BA group from T0 (M = 21.59, SE = 0.43; CI [20.75–22.44]) to T1 (M = 23.43, SE = 0.48; CI [22.48–24.38]; *M*_Diff_ = 1.84 *p* < 0.001, d = 0.35) but also, albeit smaller, an increase from T0 to T2 (M_T2_ = 23.01, SE_T2_ = 0.52; CI [21.98–24.04]; *M*_DiffT0-T2_ = 1.42 *p* < 0.001, d = 0.27; *M*_DiffT1-T2_ = − 0.42 *p* = 0.055, d = 0.43). Prosocial behavior also increased in the NO BA from T0 to T2 (M_T0_ = 21.97, SE_T0_ = 0.85; CI [20.31–23.63]; M_T1_ = 24.22, SE_T1_ = 1.04; CI [22.18–26.26]; M_T2_ = 22.99, SE_T2_ = 2.09; CI [18.75–27.24]; *M*_DiffT0-T1_ = 2.25 *p* = 0.09; *M*_DiffT0-T2_ = 1.02 *p* = 0.65; *M*_DiffT1-T2_ = − 1.23 *p* = 0.60) but such increase was not significant. As expected, the NOR group demonstrated stable prosocial behavior across all time points (M_T0_ = 27.69, SE_T0_ = 0.17; CI [27.35–28.02]; M_T1_ = 27.85, SE_T1_ = 0.18; CI [27.49–28.21]; M_T2_ = 27.98, SE_T2_ = 0.20; CI [27.58–28.38]; *M*_DiffT0-T1_ = 0.17 *p* = 0.51; *M*_DiffT0-T2_ = 0.30 *p* = 0.26; *M*_DiffT1-T2_ = 0.13 *p* = 0.64). Figure 2 illustrates such trajectories. For the NOR x NO BA group, there was no such interaction effect. Model 2, however, indicated no interaction effects for any of the groups.

Exploratory analyses regarding initial symptom severity revealed no three-way interaction between group (BA, NO BA), time (T0, T1, T2) and median group (low vs. high) for prosocial behavior (S7 and S8).

### Prediction of symptom improvement by probable parental disorder, stress, motivation, child sex and age in the BA group

Regression analyses demonstrated that only the values of the CBCL and DISYPS-prosocial behavior predicted improvements (i.e. higher values for higher improvements) in disruptive behavior problems as well as an improvement of prosocial behavior. All other variables did not predict reduction in disruptive behavior problems or improvement of prosocial behavior (see Table 3).

**Table 3:** Multiple regression analyses of probable parental disorder, motivation, stress as well as child age and sex on the CBCL (disruptive behavior—only externalizing behavior scale) and the competence scale (prosocial behavior) of the DISYPS in the BA group

	Coefficients			*t*	*p*	Collinearity	ANOVA
	*β*	SE	*β*			*VIF*	*p*
*CBCL—disruptive behavior—Model 1*							.001
(Constant)	− 2.55	1.78	–	− 1.43	.154	–	
CBCL—disruptive behavior -T0	0.34	0.08	0.38	4.37	<.001	1.00	
*CBCL—disruptive behavior—Model 2*							.001
(Constant)	6.66	7.10	–	0.94	.351	–	
CBCL—disruptive behavior -T0	0.42	0.09	0.46	4.69	<.001	1.28	
Average PMI total T0	− 1.65	1.57	− 0.10	− 1.05	.296	1.09	
Average SCL-K-9 T0	− 1.75	1.15	− 0.16	− 1.52	.131	1.42	
Probable parental disorder (CID-S)	1.06	1.46	0.07	0.73	.470	1.24	
Sex of child	− 0.86	1.45	− 0.05	− 0.59	.557	1.03	
Age of child at screening	− 0.19	0.35	− 0.05	0.55	.581	1.05	
*DISYPS–prosocial behavior—Model 1*							.001
(Constant)	− 0.58	0.15	–	− 3.88	<.001	–	
Prosocial behavior—T0	0.28	0.08	0.29	3.26	.001	1.00	
*DISYPS-prosocial behavior—Model 2*							.044
(Constant)	− 0.85	0.42	–	− 2.03	.045	–	
Prosocial behavior T0	0.30	0.09	0.32	3.41	<.001	1.08	
Average PMI total T0	0.02	0.08	0.03	0.31	.756	1.07	
Average SCL-K-9 T0	0.07	0.06	0.13	1.25	.215	1.30	
Probable parental disorder (CID-S)	0.03	0.08	0.04	0.39	.695	1.27	
Sex of child	− 0.001	0.08	− 0.001	− 0.01	.990	1.02	
Age of child at screening	0.01	0.02	0.03	0.32	.752	1.05	

Standard error per Bootstrapping (*n* = 1000). BA = children who receiving a recommendation for a prevention program after screening at the pediatrician’s office and participated in the prevention program “Baghira”; T0: Pre-intervention; CBCL—disruptive behavior = externalizing behavior scale of the Child Behavior Checklist [58]; DISYPS—prosocial behavior = Diagnostic System for Mental Disorders in Childhood and Adolescence [59]; PMI = Parental Motivation Inventory [66]; SCL-K-9 = Symptom-Checklist-Short version-9 [62]; CID-S = Composite International Diagnostic-Screener [63]. Regarding child’s sex, females were coded as 1, males as 2. A probable parental disorder was coded as 1

## Discussion

The present study, as part of a larger project, investigated the effectiveness of the child-centered, indicated prevention program "Baghira" in children from the general population who, were identified with disruptive behavior problems using the Strength and Difficulties Questionnaire (SDQ; [53]) and received a recommendation from their pediatrician to participate in the "Baghira" prevention program. Effectiveness in terms of disruptive and prosocial behavior was assessed based on parental ratings. Furthermore, the study explored the impact of child age and sex, as well as a probable parental disorder, motivation and stress on the outcome of the prevention program.

As expected, the data showed a reduction in disruptive behavior problems as measured by the CBCL across all groups. This reduction was not only evident immediately after the intervention (T1), but also 6-months later (T2). This is in line with previous research in clinical samples demonstrating a decrease of disruptive behavior problems after a social competence training [31, 32]. At baseline (T0), the BA group already differed from the NO BA group in terms of disruptive behavior problems measured by the CBCL. This difference is noteworthy because both groups should theoretically not differ, as they were identified from the general population using identical methods and cut-off values and received a recommendation for the same prevention program. The fact that children in the BA group initially exhibited a higher baseline level of disruptive behavior compared to children in the NO-BA group may explain the lower utilization of the prevention program by the latter. Those children, who participated, may have been more burdened due to more severe symptoms, leading to the higher utilization of the indicated prevention program “Baghira”. Additionally, this suggests that the combination of chosen cut-off values and pediatrician's recommendation may have been too sensitive to align with the family's perception of the necessity and potential benefits in relation to the reported and assessed negative consequences of the behavior. This is conceivable since the recommendation was based on the SDQ [53], a sensitive instrument to screen for mental problems and disorders [71], which correlates with both the CBCL—disruptive behavior and the DISYPS—prosocial behavior (Spearman correlation—CBCL—disruptive behavior: r = 0.31, *p* < 0.001; DISYPS—prosocial behavior: r = − 0.34, *p* < 0.001). However, it does not include as many items specific for disruptive behavior problems like items on disobedience as, for example, the CBCL does [58, 72].

This idea is further supported by the reasons, parents of the NO BA group provided for declining to participate in “Baghira” (see S3). For example, some families did not consider the training to be necessary for their child. It is conceivable that the parents have overestimated their child’s disruptive behavior at the pediatrician's office when using the SDQ.

In addition, we hypothesized that the BA group would converge with the NOR group in the expression of their behavior. While a trend can be observed in the trajectories from T0 to T2 for disruptive behavior as measured by the CBCL, the difference between the groups remains significant. This highlights that the BA group still exhibits distinct behavioral characteristics despite the observed improvement after participation in the indicated prevention program “Baghira”. Both, the NO BA group and the BA group showed reductions in disruptive behavior problems from T0 to T2. It is conceivable that the screening itself had an effect on families, fostering a more sensitive view of their child’s symptoms in the NO BA group. As a result, families may have initiated their own interventions, such as introducing more daily structure or making other adjustments to their everyday routines. Moreover, a selection bias might have occurred. Families in the NO BA group may have had greater resources or a different perception of the problem behavior, enabling them to address it without external support. This could explain the observed decrease over time. Although such results suggest an improvement in disruptive behavior following the participation of “Baghira”, future studies with a more rigorous and well-controlled design outside the Covid-19 pandemic are needed to determine the specific positive effects of “Baghira” as an indicated prevention program.

Furthermore, data revealed an increase in prosocial behavior as measured by the DISYPS (competence scale) in the BA group at time points T1 and T2 indicating an enduring positive effect of this indicated prevention program. Important to note, the questionnaire was completed by parents, who based their answers on their child’s everyday behavior, not on their behavior during the 90-min prevention session. Improvement of prosocial behavior by building a set of social skills is particularly important for children with disruptive behavior problems or disorders, as a deficiency of these skills is often linked to peer rejection [73]. It therefore supports them in being able to pass relevant developmental milestones, such as building friendships. Children from the BA group demonstrated a lower level of prosocial behavior than the children of the NO BA group at T0. This difference is also evident during the course of the intervention and after its completion and raises the same possible explanations as for the CBCL—disruptive behavior. Again, children in the BA group improved but did not reach the level of prosocial competence of the NOR group. Additional analyses considering initial symptom severity for both disruptive behavior problems as well as prosocial behavior showed that children with high initial symptom severity improved over time, suggesting that baseline symptom severity plays an important role in symptom change trajectories. However, this effect was not specific to the BA group, indicating that other non-specific factors such as increased parent awareness due to the screening at the pediatrician’s office being at play. The convergence between the high and low symptom trajectories as observed in the BA group may nonetheless indicate a compensatory effect of program participation. As the median-split model was exploratory in nature, further research using matched or stratified designs is necessary. Further, the NO BA group is also characterized by a large variance of scores for all outcome measures and a small number of total sample size. The extent of disruptive behavior problems varies widely among the few children in this group. This raises the question of representativeness making further research necessary that directly compares a sufficient number of children who participate in a prevention program and those who refuse participation.

In order to gain more information about essential factors that are predictive for an improvement in symptoms after participation in an indicated prevention program such as “Baghira”, we investigated a probable parental disorder, motivation, stress as well as child age and sex. First, it is encouraging that neither the age nor the sex of the child had any effect on the improvement of disruptive behavior problems in the BA group. Regarding sex, this resonates with previous findings in clinical samples [42, 74] but in relation to the child’s age, the results are in contrast to our hypotheses.”Baghira”, with the present adaptations, is suitable for both sexes and even younger children and shows a decrease in disruptive behavior problems, as shown for the *Baghira training* [40]*.*

Within intention-to-treat analyses regarding parental factors, data did not reveal any predictive quality of probable parental disorder or stress for the improvement of disruptive behavior problems in children although these factors have previously been found to play an essential role for children’s and parents’ participation in clinical and/or preventive interventions [51, 66, 75]. Studies have further shown that parents with mental health problems often find it more difficult to participate in such interventions with their child and to support the child in implementing what has been learned in everyday life [47, 50]. Conceivably, parents with mental health issues and elevated stress levels, both of which are also linked to motivation, could be underrepresented in the current sample, as they may have opted out of participation by choosing not to have the SDQ completed at the pediatrician’s office. However, this also resonates with a previous finding showing no association between maternal depression and treatment outcome in offspring participating in an indicated prevention program [76]. Additionally, parental motivation also did not seem to impact the training results in the BA group. It should be noted, however, that the program that was used within the scope of this study is child-centered in nature. Effects of parent motivation may well be present in parent-centered preventive and clinical programs [51] but may also be an indicator of the child’s symptom recurrence as shown in one study on a clinical sample [77].

A key advantage of the PROMPt project is its relatively low-threshold integration into routine pediatric care, making accessible for families, who might otherwise have difficulty obtaining appropriate support services. Research indicates that children whose parents have mental health problems are nearly twice as likely to experience mental health problems themselves compared to those with parents without such problems [78]. In this context, the PROMPt project certainly has the potential to offer all parents with low-threshold access to support services as needed. Furthermore, “Baghira” appears to have a lasting impact, with effects not only observed not only immediately after the training, but also up to 6 months later. Thus, the present study is an important addition to the existing literature, as longitudinal studies of training effects on disruptive behavior problems in children that are child focused within a prevention context are scarce [79, 80].

### Limitations

This study has limitations that should be acknowledged. Although the sample can be considered representative of other urban areas in Germany, there were differences in household income between the groups. Furthermore, the data revealed that families with a higher household income were more likely to participate in the project. Consequently, a selection bias—commonly found in epidemiological studies on child and adolescent (mental) health—may also have influenced the findings [81]. However, as previously demonstrated by Weniger and colleagues, household income was only associated with participation in the on-site initial interview with a member of the study team, but not with the actual participation in the prevention program [52]. This discrepancy might be related to the participating medical practices, as project participation was voluntary, and the catchment areas—along with the socio-economic status of the families—vary between practices, leading to the observed effect. The study was conducted within the German healthcare system using the internationally validated SDQ as a screening measure for recognition of behavior problems. However, transferability to other healthcare systems (e.g., insurance models, funding structures) or cultural contexts should be examined in future studies. Moreover, the study was conducted during the Covid-19 pandemic, which has been shown to adversely affect children’s mental health [82]. The pandemic and its associated constraints, such as contact restrictions, made it more difficult for children and families to integrate the strategies they have learned directly into their daily lives, potentially reducing the preventive effect. This may also serve as an explanation for the lack of greater convergence between the BA and NOR groups, though further research is needed. Although this study was not a randomized controlled trial, it adds to the literature as it demonstrates effects of targeted allocation to an indicated prevention program through the pediatrician’s office. This is important because the utilization rates for indicated prevention programs tend to be low [83]. However, to the best of our knowledge, no specific data on the utilization rates regarding disruptive behavior problems are available. In addition, current data of this study do not allow for conclusions about the extent to which the actual risk of developing a disorder in the course of the child’s life has been reduced. Further research that integrates additional time points and instruments to assess mental disorders is therefore necessary, particularly in light of the potential cost-effectiveness of the intervention. Last, because the available data gained in this study are based solely on parental judgment. This parental response bias may have led parents to rate their children more favorably after participating in the indicated prevention program “Baghira”. This needs to be kept in mind when interpreting current results. Future studies should therefore also incorporate questionnaires that reflect children’s judgment.

## Conclusion

The results of the PROMPt study showed a reduction in disruptive behavior problems following participation in the indicated prevention program “Baghira,” even over a longer period. On the one hand, due to certain limiting circumstances of the study, these findings should serve as a stimulus for further research into the identification of children at risk for mental health issues within routine pediatric care and their subsequent referral to preventive services. On the other hand, the program should already be made available to as many children and adolescents as possible who are identified with disruptive behavior problems during routine pediatric check-ups.

## Supplementary Information

Supplementary Material 1.

## Data Availability

The data that support the findings of this study are available from the corresponding author upon reasonable request.

## References

[1] Klipker K, Baumgarten F, Göbel K, Lampert T, Hölling H. Psychische Auffälligkeiten bei Kindern und Jugendlichen in Deutschland– Querschnittergebnisse aus KiGGS Welle 2 und Trends. J Health Monit. 2018;3(3):37–45. 10.17886/RKI-GBE-2018-077.

[2] Copeland WE, Wolke D, Shanahan L, Costello EJ. Adult functional outcomes of common childhood psychiatric problems: a prospective, longitudinal study. JAMA Psychiat. 2015;72(9):892. 10.1001/jamapsychiatry.2015.0730. 10.1001/jamapsychiatry.2015.0730 PMC4706225 26176785

[3] Mulraney M, Coghill D, Bishop C, Mehmed Y, Sciberras E, Sawyer M, Efron D, Hiscock H. A systematic review of the persistence of childhood mental health problems into adulthood. Neurosci Biobehav Rev. 2021;129:182–205. 10.1016/j.neubiorev.2021.07.030. 34363845 10.1016/j.neubiorev.2021.07.030

[4] Sacco R, Camilleri N, Eberhardt J, Umla-Runge K, Newbury-Birch D. A systematic review and meta-analysis on the prevalence of mental disorders among children and adolescents in Europe. Eur Child Adolesc Psychiatry. 2022. 10.1007/s00787-022-02131-2. 36581685 10.1007/s00787-022-02131-2 PMC9800241

[5] Ravens-Sieberer U, Wille N, Erhart M, Bettge S, Wittchen H-U, Rothenberger A, Herpertz-Dahlmann B, Resch F, Hölling H, Bullinger M, Barkmann C, Schulte-Markwort M, Döpfner M, as the BELLA study group. Prevalence of mental health problems among children and adolescents in Germany: results of the BELLA study within the National Health Interview and Examination Survey. Eur Child Adolesc Psychiatry. 2008;17(S1):22–33. 10.1007/s00787-008-1003-2. 19132301 10.1007/s00787-008-1003-2

[6] Ewest F, Reinhold T, Vloet TD, Wenning V, Bachmann CJ. Durch Jugendliche mit Störungen des Sozialverhaltens ausgelöste Krankenkassenausgaben: Eine gesundheitsökonomische Analyse von Versichertendaten einer gesetzlichen Krankenkasse. Kindheit Entwicklung. 2013;22(1):41–7. 10.1026/0942-5403/a000097.

[7] Fatori D, Salum G, Itria A, Pan P, Alvarenga P, Rohde LA, Bressan R, Gadelha A, de Jesus Mari J, Conceição do Rosário M, Manfro G, Polanczyk G, Miguel EC, Graeff-Martins AS. The economic impact of subthreshold and clinical childhood mental disorders. J Ment Health. 2018;27(6):588–94. 10.1080/09638237.2018.1466041. 29708045 10.1080/09638237.2018.1466041

[8] Kuschel A, Heinrichs N, Bertram H, Naumann S, Hahlweg K. Psychische Auffälligkeiten bei Kindergartenkindern aus der Sicht der Eltern und Erzieherinnen in Abhängigkeit von soziodemografischen Merkmalen. Kindheit und Entwicklung. 2008;17(3):161–72. 10.1026/0942-5403.17.3.161.

[9] Odgers CL, Moffitt TE, Broadbent JM, Dickson N, Hancox RJ, Harrington H, Poulton R, Sears MR, Thomson WM, Caspi A. Female and male antisocial trajectories: from childhood origins to adult outcomes. Dev Psychopathol. 2008;20(2):673–716. 10.1017/S0954579408000333. 18423100 10.1017/S0954579408000333

[10] Zahrt DM, Melzer-Lange MD. Aggressive behavior in children and adolescents. Pediatr Rev. 2011;32(8):325–32. 10.1542/pir.32.8.325. 21807873 10.1542/pir.32-8-325

[11] Petermann F, Helmsen J, Koglin U. Expansive Verhaltensstörungen. Monatsschrift Kinderheilkunde. 2010;158(1):22–7. 10.1007/s00112-009-2057-z.

[12] Becker A, Roessner V, Breuer D, Döpfner M, Rothenberger A. Relationship between quality of life and psychopathological profile: data from an observational study in children with ADHD. Eur Child Adolesc Psychiatry. 2011;20(S2):267–75. 10.1007/s00787-011-0204-2. 10.1007/s00787-011-0204-2 PMC3180591 21901415

[13] Jia M, Mikami AY. Peer preference and friendship quantity in children with externalizing behavior: distinct influences on bully status and victim status. J Abnorm Child Psychol. 2015;43(5):957–69. 10.1007/s10802-014-9956-8. 25411126 10.1007/s10802-014-9956-8

[14] Otto C, Haller A-C, Klasen F, Hölling H, Bullinger M, Ravens-Sieberer U, on behalf of the BELLA study group. Risk and protective factors of health-related quality of life in children and adolescents: results of the longitudinal BELLA study. PLoS ONE. 2017;12(12): e0190363. 10.1371/journal.pone.0190363. 29284054 10.1371/journal.pone.0190363 PMC5746247

[15] Schlack R, Peerenboom N, Neuperdt L, Junker S, Beyer A-K. Effekte psychischer Auffälligkeiten in Kindheit und Jugend im jungen Erwachsenenalter: Ergebnisse der KiGGS-Kohorte. 2021. 10.25646/8862.

[16] Sturaro C, van Lier PAC, Cuijpers P, Koot HM. The role of peer relationships in the development of early school-age externalizing problems: peer relationships and externalizing. Child Dev. 2011;82(3):758–65. 10.1111/j.1467-8624.2010.01532.x. 21410917 10.1111/j.1467-8624.2010.01532.x

[17] Asselmann E, Wittchen H-U, Lieb R, Beesdo-Baum K. Sociodemographic, clinical, and functional long-term outcomes in adolescents and young adults with mental disorders. Acta Psychiatr Scand. 2018;137(1):6–17. 10.1111/acps.12792. 28861892 10.1111/acps.12792

[18] Olson SL, Choe DE, Sameroff AJ. Trajectories of child externalizing problems between ages 3 and 10 years: contributions of children’s early effortful control, theory of mind, and parenting experiences. Dev Psychopathol. 2017;29(4):1333–51. 10.1017/S095457941700030X. 28290269 10.1017/S095457941700030X PMC11227349

[19] DSM-5 American Psychiatric Association. Diagnostic and statistical manual of mental disorders. Arlington: American Psychiatric Publishing; 2013.

[20] Haller A-C, Klasen F, Petermann F, Barkmann C, Otto C, Schlack R, Ravens-Sieberer U. Langzeitfolgen externalisierender Verhaltensauffälligkeiten: Ergebnisse der BELLA-Kohortenstudie. Kindheit Entwicklung. 2016;25(1):31–40. 10.1026/0942-5403/a000186.

[21] Görtz-Dorten A, Hanisch C, Hautmann C, Döpfner M. Prävention externaler Störungen–zum Stand der Forschung. Z Kinder Jugendpsychiatr Psychother. 2020;48(6):459–68. 10.1024/1422-4917/a000650. 30882267 10.1024/1422-4917/a000650

[22] Conti G, Heckman JJ. The developmental approach to child and adult health. Pediatrics. 2013;131(Supplement_2):S133–41. 10.1542/peds.2013-0252d. 23547057 10.1542/peds.2013-0252d PMC4075134

[23] Acland EL, Jambon M, Malti T. Children’s emotion recognition and aggression: a multi-cohort longitudinal study. Aggressive Behav. 2021;47(6):646–58. 10.1002/ab.21989. 10.1002/ab.21989 34369593

[24] Arango C, Díaz-Caneja CM, McGorry PD, Rapoport J, Sommer IE, Vorstman JA, McDaid D, Marín O, Serrano-Drozdowskyj E, Freedman R, Carpenter W. Preventive strategies for mental health. Lancet Psychiatry. 2018;5(7):591–604. 10.1016/S2215-0366(18)30057-9. 29773478 10.1016/S2215-0366(18)30057-9

[25] Ortelbach N, Bovenschen I, Gerlach J, Peter C, Scheithauer H. Design, implementation, and evaluation of a preventive intervention program to foster social-emotional development and attachment security of toddlers in early childhood education and care: The Papilio-U3 Program. Int J Dev Sustain. 2023;16(3–4):63–79. 10.3233/DEV-220336.

[26] Scheithauer H, Scheer H. Developmentally appropriate prevention of behavioral and emotional problems, social-emotional learning, and developmentally appropriate practice for early childhood education and care—The Papilio Approach from 0 to 9. Int J Dev Sustain. 2023;16(3–4):57–62. 10.3233/DEV-220337.

[27] Beelmann A, Pfost M, Schmitt C. Prävention und Gesundheitsförderung bei Kindern und Jugendlichen: Eine Meta-Analyse der deutschsprachigen Wirksamkeitsforschung. Zeitschrift für Gesundheitspsychologie. 2014;22(1):1–14. 10.1026/0943-8149/a000104.

[28] Wilson SJ, Lipsey MW. School-based interventions for aggressive and disruptive behavior. Am J Prev Med. 2007;33(2):S130–43. 10.1016/j.amepre.2007.04.011. 17675014 10.1016/j.amepre.2007.04.011 PMC2246021

[29] Le LK-D, Esturas AC, Mihalopoulos C, Chiotelis O, Bucholc J, Chatterton ML, Engel L. Cost-effectiveness evidence of mental health prevention and promotion interventions: a systematic review of economic evaluations. PLoS Med. 2021;18(5): e1003606. 10.1371/journal.pmed.1003606. 33974641 10.1371/journal.pmed.1003606 PMC8148329

[30] Reichle B, Roth I. Beziehungsorientierte Intervention am Beispiel des „Ich bleibe cool“-Trainings zur Förderung prosozialer Verhaltensweisen und konstruktiver Konfliktlösestrategien bei Kindern im Grundschulalter. Prax Kinderpsychol Kinderpsychiatr. 2007;56(5):463–82. 10.13109/prkk.2007.56.5.463. 17725186 10.13109/prkk.2007.56.5.463

[31] Giudice TD, Lindenschmidt T, Hellmich M, Hautmann C, Döpfner M, Görtz-Dorten A. Stability of the effects of a social competence training program for children with oppositional defiant disorder/conduct disorder: a 10-month follow-up. Eur Child Adolesc Psychiatry. 2022. 10.1007/s00787-021-01932-1. 35279770 10.1007/s00787-021-01932-1 PMC10460314

[32] Görtz-Dorten A, Benesch C, Berk-Pawlitzek E, Faber M, Hautmann C, Hellmich M, Lindenschmidt T, Schuh L, Stadermann R, Doepfner M. Efficacy of individualized social competence training for children with oppositional defiant disorders/conduct disorders: a randomized controlled trial with an active control group. Eur Child Adolesc Psychiatry. 2019;28(2):165–75. 10.1007/s00787-018-1144-x. 29594368 10.1007/s00787-018-1144-x

[33] Görtz-Dorten A, Döpfner M. Therapieprogramm für Kinder mit aggressivem Verhalten (THAV). Hogrefe Verlag;2019.

[34] Aebi M, Perriard R, Scherrer BS, Wettach R. *Kinder mit oppositionellem und aggressivem Verhalten: Das Baghira-Training.* Hogrefe Verlag GmbH & Company KG; 2012.

[35] Goodman R. Psychometric properties of the strengths and difficulties questionnaire. J Am Acad Child Adolesc Psychiatry. 2001;40(11):1337–45. 10.1097/00004583-200111000-00015. 11699809 10.1097/00004583-200111000-00015

[36] Seeling S, Prütz F, Gutsche J. Utilization of paediatric and general medical services by children and adolescents in Germany. Results of the cross-sectional KiGGS Wave 2 study and trends. 2018. 10.17886/RKI-GBE-2018-099. 10.17886/RKI-GBE-2018-099 PMC8852780 35586147

[37] Weniger M, Beesdo-Baum K, Roessner V, Hense H, Knappe S. Wie gelingt die Prävention psychischer Beschwerden?: Von der Vorsorgeuntersuchung zur indikativen Präventionsmaßnahme bei emotionalen und Verhaltensauffälligkeiten im Vor- und Grundschulalter: Eine prospektive Implementationsstudie. Prävention und Gesundheitsförderung. 2022;17(1):75–82. 10.1007/s11553-021-00838-9. 10.1007/s11553-021-00838-9 PMC7934121 40477285

[38] Hunnikin LM, Wells AE, Ash DP, Van Goozen SHM. The nature and extent of emotion recognition and empathy impairments in children showing disruptive behaviour referred into a crime prevention programme. Eur Child Adolesc Psychiatry. 2020;29(3):363–71. 10.1007/s00787-019-01358-w. 31154516 10.1007/s00787-019-01358-w PMC7056692

[39] Sanders MR. Triple P-Positive Parenting Program: towards an empirically validated multilevel parenting and family support strategy for the prevention of behavior and emotional problems in children. Clin Child Fam Psychol Rev. 1999;2(2):71–90. 10.1023/A:1021843613840. 11225933 10.1023/a:1021843613840

[40] Wettach R, Aebi M. Pilotstudie zur Wirksamkeit einer multimodalen Gruppenbehandlung für Kinder mit einer Störung des Sozialverhaltens mit oppositionellem, aufsässigem Verhalten in der klinischen Grundversorgung. Z Kinder Jugendpsychiatr Psychother. 2016;44(3):220–30. 10.1024/1422-4917/a000424. 27216328 10.1024/1422-4917/a000424

[41] Grasmann D, Stadler C. VIA–Intensivtherapeutischer Behandlungsansatz bei Störungen des Sozialverhaltens. Z Kinder Jugendpsychiatr Psychother. 2011;39(1):23–31. 10.1024/1422-4917/a000080. 21267948 10.1024/1422-4917/a000080

[42] Ollendick TH, Greene RW, Austin KE, Fraire MG, Halldorsdottir T, Allen KB, Jarrett MA, Lewis KM, Whitmore Smith M, Cunningham NR, Noguchi RJP, Canavera K, Wolff JC. Parent management training and collaborative & proactive solutions: a randomized control trial for oppositional youth. J Clin Child Adolesc Psychol. 2016;45(5):591–604. 10.1080/15374416.2015.1004681. 25751000 10.1080/15374416.2015.1004681 PMC4564364

[43] Forehand R, Lafko N, Parent J, Burt KB. Is parenting the mediator of change in behavioral parent training for externalizing problems of youth? Clin Psychol Rev. 2014;34(8):608–19. 10.1016/j.cpr.2014.10.001. 25455625 10.1016/j.cpr.2014.10.001 PMC4254490

[44] Ackermann K, Büttner G, Bernhard A, Martinelli A, Freitag CM, Schwenck C. Freundschaftsqualitäten und unterschiedliche Formen aggressiven Verhaltens bei Jungen und Mädchen im späten Kindes- und Jugendalter. Kindheit Entwicklung. 2018;27(2):81–90. 10.1026/0942-5403/a000248.

[45] Connell AM, Goodman SH. The association between psychopathology in fathers versus mothers and children’s internalizing and externalizing behavior problems: a meta-analysis. Psychol Bull. 2002;128(5):746–73. 10.1037/0033-2909.128.5.746. 12206193 10.1037/0033-2909.128.5.746

[46] Goodman SH, Rouse MH, Connell AM, Broth MR, Hall CM, Heyward D. Maternal depression and child psychopathology: a meta-analytic review. Clin Child Fam Psychol Rev. 2011;14(1):1–27. 10.1007/s10567-010-0080-1. 21052833 10.1007/s10567-010-0080-1

[47] Muratori P, Milone A, Nocentini A, Manfredi A, Polidori L, Ruglioni L, Lambruschi F, Masi G, Lochman JE. Maternal depression and parenting practices predict treatment outcome in Italian children with disruptive behavior disorder. J Child Fam Stud. 2015;24(9):2805–16. 10.1007/s10826-014-0085-3.

[48] Dedousis-Wallace A, Drysdale SA, McAloon J, Ollendick TH. Parental and familial predictors and moderators of parent management treatment programs for conduct problems in youth. Clin Child Fam Psychol Rev. 2021;24(1):92–119. 10.1007/s10567-020-00330-4. 33074467 10.1007/s10567-020-00330-4

[49] Păsărelu C-R, Dobrean A, Florean IS, Predescu E. Parental stress and child mental health: a network analysis of Romanian parents. Curr Psychol. 2023;42(28):24275–87. 10.1007/s12144-022-03520-1. 10.1007/s12144-022-03520-1 PMC9362691 35967498

[50] Haine-Schlagel R, Dickson KS, Shapiro AF, May GC, Cheng P. Parent mental health problems and motivation as predictors of their engagement in community-based child mental health services. Child Youth Serv Rev. 2019;104: 104370. 10.1016/j.childyouth.2019.06.005. 31258235 10.1016/j.childyouth.2019.06.005 PMC6599621

[51] Pereira AI, Barros L. Parental cognitions and motivation to engage in psychological interventions: a systematic review. Child Psychiatry Hum Dev. 2019;50(3):347–61. 10.1007/s10578-018-0852-2. 30430390 10.1007/s10578-018-0852-2

[52] Weniger M, Beesdo-Baum K, Ernst J, Siegmund CB, Porst PT, McDonald M, Roessner V, Knappe S. Indikative Präventionsprogramme zur Förderung der seelischen Gesundheit im Vor- und Grundschulalter: Teilnahmebereitschaft von Kinderärzt*innen und Familien an einer innovativen Versorgungskette. Bundesgesundheitsblatt - Gesundheitsforschung - Gesundheitsschutz. 2024;67(1):23–35. 10.1007/s00103-023-03787-0. 37921872 10.1007/s00103-023-03787-0 PMC10776478

[53] Goodman R. The strengths and difficulties questionnaire: a research note. J Child Psychol Psychiatry. 1997;38(5):581–6. 10.1111/j.1469-7610.1997.tb01545.x. 9255702 10.1111/j.1469-7610.1997.tb01545.x

[54] Siegmund CB, Zink J, Porst PT, Weniger M, Knappe S, McDonald M, Roessner V, Beesdo-Baum K. Disruptive behavior and emotional problems in children screened in routine health care: prevalence and effectiveness of indicated prevention. Submitted. 10.1007/s10802-024-01221-w PMC11461602 38963517

[55] Zink J, Weniger M, Porst PT, Siegmund CB, McDonald M, Rückert F, Roessner V, Knappe S, Beesdo-Baum K. Indicated prevention for children screened in routine health care: effectiveness of a social skills program on social anxiety and depressive symptoms. Res Child Adolesc Psychopathol. 2024. 10.1007/s10802-024-01221-w. 38963517 10.1007/s10802-024-01221-w PMC11461602

[56] Ahrens-Eipper S, Leplow B, Nelius K. Mutig werden mit Til Tiger: Ein Trainingsprogramm für sozial unsichere Kinder. (2., erweiterte Auflage). Hogrefe;2010.

[57] Achenbach TM. Manual for the CBCL/4-18 and 1991 Profile. Burlington, VT: University of Vermont; 1991.

[58] Arbeitsgruppe Deutsche Child Behavior Checklist. Elternfragebogen über das Verhalten von Kindern und Jugendlichen; deutsche Bearbeitung der Child Behavior Checklist (CBCL/4- 18). Einführung und Anleitung zur Handauswertung (2. Auflage mit deutschen Normen). Arbeitsgruppe Kinder-, Jugend- und Familiendiagnostik. 1998.

[59] Döpfner M, Görtz-Dorten A, Lehmkuhl G. Diagnostik-System für psychische Störungen nach ICD-10 und DSM-5 für Kinder und Jugendliche–III (DISYPS-III). Göttingen, Germany: Hogrefe; 2017.

[60] Siegmund CB, Zink J, Porst PT, Weniger M, McDonald M, Knappe S, Roessner V, Beesdo-Baum K. Effectiveness comparison of an indicated child-centered group prevention program for disruptive behavior problems in children with vs. without co-occurring emotional problems. Submitted. 10.1186/s40359-025-03392-7 PMC12418713 40926265

[61] Döpfner M, Schmeck K, Berner W. Handbuch: Elternfragebogen über das Verhalten von Kindern und Jugendlichen. Forschungsergebnisse zur deutschen Fassung der Child Behavior Checklist (CBCL). Arbeitsgruppe Kinder-, Jugend- und Familiendiagnostik. 1994.

[62] Klaghofer R, Brähler E. Konstruktion und Teststatistische Prüfung einer Kurzform der SCL-90–R [Construction and test statistical evaluation of a short version of the SCL-90–R]. Z Klin Psychol Psychiatr Psychother. 2001;49(2):115–24.

[63] Wittchen H-U, Höfler M, Gander F, Pfister H, Storz S, Bedirhan Ü, Müller N, Kessler RC. Screening for mental disorders: performance of the composite international diagnostic—Screener (CID–S). Int J Methods Psychiatr Res. 1999;8(2):59–70.

[64] Petrowski K, Schmalbach B, Kliem S, Hinz A, Brähler E. Symptom-Checklist-K-9: Norm values and factorial structure in a representative German sample. PLOS ONE, 2019;14(4):1–16. 10.1371/journal.pone.0213490 10.1371/journal.pone.0213490 PMC6450622 30951538

[65] Wittchen H-U, Boyer P. Screening for anxiety disorders: sensitivity and specificity of the anxiety screening questionnaire (ASQ-15). Br J Psychiatry. 1998;173(Suppl. 34):10–7. 9829011

[66] Wittchen H-U, Nelson GB, Lachner G. Prevalence of mental disorders and psychosocial impairments in adolescents and young adults. Psychol Med. 1998;28:109–26. 9483687 10.1017/s0033291797005928

[67] Nock MK, Photos V. Parent motivation to participate in treatment: assessment and prediction of subsequent participation. J Child Fam Stud. 2006;15(3):333–46. 10.1007/s10826-006-9022-4.

[68] IBM Corp. IBM SPSS Statistics für Windows (Version 29.0.0.0) [Computer software]. IBM Corp;2022.

[69] R Core Team. R: a language and environment for statistical computing. R Foundation for Statistical Computing [Computer software]. https://www.R-project.org/. 2021.

[70] Field A. Discovering statistics using IBM SPSS statistics. 5th ed. Thousand Oaks: SAGE; 2018.

[71] Nielsen LG, Rimvall MK, Clemmensen L, Munkholm A, Elberling H, Olsen EM, Rask CU, Skovgaard AM, Jeppesen P. The predictive validity of the Strengths and Difficulties Questionnaire in preschool age to identify mental disorders in preadolescence. PLoS ONE. 2019;14(6): e0217707. 10.1371/journal.pone.0217707. 31158249 10.1371/journal.pone.0217707 PMC6546211

[72] Mansolf M, Blackwell CK, Cummings P, Choi S, Cella D. Linking the child behavior checklist to the strengths and difficulties questionnaire. Psychol Assess. 2022;34(3):233–46. 10.1037/pas0001083. 34843282 10.1037/pas0001083 PMC9718585

[73] Glenn DE, Michalska KJ, Lee SS. Social skills moderate the time-varying association between aggression and peer rejection among children with and without ADHD. Aggressive Behav. 2021;47(6):659–71. 10.1002/ab.21991. 10.1002/ab.21991 PMC10155268 34426990

[74] Hautmann C, Eichelberger I, Hanisch C, Plück J, Walter D, Döpfner M. The severely impaired do profit most: short-term and long-term predictors of therapeutic change for a parent management training under routine care conditions for children with externalizing problem behavior. Eur Child Adolesc Psychiatry. 2010;19(5):419–30. 10.1007/s00787-009-0072-1. 19915886 10.1007/s00787-009-0072-1

[75] Sun J, Singletary B, Jiang H, Justice LM, Lin T-J, Purtell KM. Child behavior problems during COVID-19: associations with parent distress and child social-emotional skills. J Appl Dev Psychol. 2022;78: 101375. 10.1016/j.appdev.2021.101375. 34924662 10.1016/j.appdev.2021.101375 PMC8668344

[76] Hanisch C, Freund-Braier I, Hautmann C, Jänen N, Plück J, Brix G, Eichelberger I, Döpfner M. Detecting Effects of the Indicated Prevention Programme for Externalizing Problem Behaviour (PEP) on child symptoms, parenting, and parental quality of life in a randomized controlled trial. Behav Cogn Psychother. 2010;38(1):95–112. 10.1017/S1352465809990440. 19995467 10.1017/S1352465809990440

[77] Nock MK, Kazdin AE. Randomized controlled trial of a brief intervention for increasing participation in parent management training. J Consult Clin Psychol. 2005;73(5):872–9. 10.1037/0022-006X.73.5.872. 16287387 10.1037/0022-006X.73.5.872

[78] Plass-Christl A, Haller A-C, Otto C, Barkmann C, Wiegand-Grefe S, Hölling H, Schulte-Markwort M, Ravens-Sieberer U, Klasen F. Parents with mental health problems and their children in a German population based sample: results of the BELLA study. PLoS ONE. 2017;12(7): e0180410. 10.1371/journal.pone.0180410. 28671981 10.1371/journal.pone.0180410 PMC5495394

[79] Smedler A-C, Hjern A, Wiklund S, Anttila S, Pettersson A. Programs for prevention of externalizing problems in children: limited evidence for effect beyond 6 months post intervention. Child Youth Care Forum. 2015;44(2):251–76. 10.1007/s10566-014-9281-y. 26696756 10.1007/s10566-014-9281-y PMC4676792

[80] Goodman A, Gatward R. Who are we missing? Area deprivation and survey participation. Eur J Epidemiol. 2008;23(6):379–87. 10.1007/s10654-008-9248-0. 18409005 10.1007/s10654-008-9248-0

[81] Kauhanen L, Wan Mohd Yunus WMA, Lempinen L, Peltonen K, Gyllenberg D, Mishina K, Gilbert S, Bastola K, Brown JSL, Sourander A. A systematic review of the mental health changes of children and young people before and during the COVID-19 pandemic. Eur Child Adolesc Psychiatry. 2023;32(6):995–1013. 10.1007/s00787-022-02060-0. 35962147 10.1007/s00787-022-02060-0 PMC9373888

[82] Bauer S, Geiger L, Niggemann R, Seidel J, Medizinischer Dienst des Spitzenverbandes Bund der Krankenkassen e.V. (MDS). Präventionsbericht 2020. Leistungen der gesetzlichen Krankenversicherung: Primärprävention und Gesundheitsförderung. Leistungen der sozialen Pflegeversicherung: Prävention in stationären Pflegeeinrichtungen. Berichtsjahr 2019 (Berichtsjahr 2019). 2020.

[83] Cohen J. Statistical power analysis for the behavioral sciences. Academic Press; 1988.

